# Mucosal and faecal neutrophil gelatinase-associated lipocalin as potential biomarkers for collagenous colitis

**DOI:** 10.1007/s00535-021-01814-y

**Published:** 2021-08-19

**Authors:** Ingunn Bakke, Gunnar Andreas Walaas, Torunn Bruland, Elin Synnøve Røyset, Atle van Beelen Granlund, Celia Escudero-Hernández, Silje Thorsvik, Andreas Münch, Arne Kristian Sandvik, Ann Elisabet Østvik

**Affiliations:** 1 Department of Clinical and Molecular Medicine (IKOM), Faculty of Medicine and Health Sciences, NTNU—Norwegian University of Science and Technology, Prinsesse Kristinas Gate 1, 7489 Trondheim, Norway; 2 Clinic of Laboratory Medicine, St. Olav’s University Hospital, Trondheim, Norway; 3 Clinic of Medicine, St. Olav’s University Hospital, Trondheim, Norway; 4 Department of Pathology, Clinic of Laboratory Medicine, St. Olav’s University Hospital, Trondheim, Norway; 5 Centre of Molecular Inflammation Research (CEMIR), Faculty of Medicine and Health Sciences, NTNU—Norwegian University of Science and Technology, Prinsesse Kristinas Gate 1, 7489 Trondheim, Norway; 6 Department of Biomedical and Clinical Sciences (BVK), Linköping University, Linköping, Sweden; 7 Department of Gastroenterology and Hepatology, Clinic of Medicine, St. Olav’s University Hospital, Trondheim, Norway; 8 Division of Gastroenterology and Hepatology, Linköping University Hospital, Linköping, Sweden; 9 Present Address: Institute of Clinical Molecular Biology (IKMB), Christian-Albrechts-University Kiel, and University Hospital Schleswig Holstein, Kiel, Germany

**Keywords:** Microscopic colitis, Chronic diarrhoea, Inflammatory bowel disease, Calprotectin, Irritable bowel syndrome

## Abstract

**Background:**

Collagenous colitis (CC) is an inflammatory bowel disease where chronic diarrhoea is the main symptom. Diagnostic markers distinguishing between CC and other causes of chronic diarrhoea remain elusive. This study explores neutrophil gelatinase-associated lipocalin (NGAL) and its mRNA lipocalin2 (*LCN2*) as histological and faecal disease markers in CC.

**Methods:**

NGAL/*LCN2* were studied in colonic biopsies from CC patients before and during budesonide treatment using RNA sequencing (*n* = 9/group), in situ hybridization (ISH) (*n* = 13–22/group) and immunohistochemistry (IHC) (*n* = 14–25/group). Faecal samples from CC (*n* = 3–28/group), irritable bowel syndrome diarrhoea (IBS-D) (*n* = 14) and healthy controls (HC) (*n* = 15) were assayed for NGAL and calprotectin.

**Results:**

NGAL/*LCN2* protein and mRNA expression were upregulated in active CC vs HC, and vs paired samples of treated CC in clinical remission. IHC and ISH localized increased NGAL/*LCN2* mainly to epithelium of active CC, compared to almost absence in HC and treated CC. In contrast, calprotectin was solely expressed in immune cells. Despite great individual differences, faecal NGAL was significantly increased in active CC compared to HC, IBS-D and treated CC and had high test sensitivity. Faecal calprotectin levels were variably increased in active CC, but the values remained below usual clinical cut-offs.

**Conclusion:**

NGAL/*LCN2* is upregulated in the epithelium of active CC and reduced during budesonide-induced clinical remission to the level of HC and IBD-S. This was reflected in NGAL faecal concentrations. We propose NGAL as an IHC marker for disease activity in CC and a potential faecal biomarker discriminating CC from HC and IBS-D.

**Supplementary Information:**

The online version contains supplementary material available at 10.1007/s00535-021-01814-y.

## Introduction

Collagenous colitis (CC) is a subgroup of microscopic colitis (MC), a chronic inflammatory bowel disease (IBD) differing clinically and pathobiologically from Crohn’s disease and ulcerative colitis, the classic IBDs. Symptoms of CC are frequent and watery diarrhoea without blood. Colonoscopy reveals no or minimal pathology, while biopsies show preserved mucosal architecture with chronic inflammation in the lamina propria, epithelial injury (flattening, focal detachments) and abnormal (> 10 µm) subepithelial depositions of collagen. Histopathology defines another subgroup of MC, lymphocytic colitis (LC), which is distinguished by enhanced lymphocytic infiltration of the surface epithelium instead of increased collagen band [1, 2]. In addition, there are variant forms of MC with incomplete histological features [1].

Similarly to the classic IBDs, aetiology and pathogenesis of MC including CC, are unknown [3, 4]. Treatment with budesonide helps many patients (~ 80%) but without universal success, and relapse after discontinuation is common [4, 5]. Non-invasive biomarkers are lacking and current definition of disease activity is based on the clinical observation of stool frequency and watery consistency [6]. Chronic diarrhoea is challenging as it is common at all ages, affecting 5% of the general population [7] with a large number of differential diagnoses including CC. Colonoscopy is mandatory for people over 50 years with changed bowel habits, but up to 25% of the CC population is younger or even children [8–10]. As a result, due to overlapping symptoms with e.g. diarrhoea-dominant irritable bowel syndrome (IBS-D), it can be difficult to select those in need of colonoscopy to confirm their CC diagnosis and to receive a potentially effective drug [11, 12]. Thus, a non-invasive faecal biomarker is of interest, both to select patients for endoscopy and to assess treatment response. Adding to the clinical challenges of CC, the initial histopathological diagnosis may also be difficult [13, 14], as there is no clear correlation between histopathological findings and clinical disease activity [15].

The most utilized biomarker for ulcerative colitis and Crohn’s disease is faecal calprotectin, which is useful particularly for diagnosis and follow-up [16, 17]. Being highly expressed in neutrophils, increased levels in faeces reflect a neutrophil-dominated inflammation like classic IBD. However, calprotectin detection is not a conclusive marker for low-level inflammatory gut disorders with more monocytic infiltration like CC [18, 19]. Neutrophil gelatinase-associated lipocalin (NGAL, gene name lipocalin 2 (*LCN2*)) is a putative faecal biomarker for ongoing inflammation in IBD [16]. NGAL is expressed in the same immune cells as calprotectin [20, 21], but differs by also being massively upregulated in colonic epithelial cells during inflammation [22, 23]. NGAL has bacteriostatic effects by inhibiting siderophore-mediated bacterial iron uptake [20, 24]. Other functions remain only partly understood, like an undefined role in cellular proliferation and mucosal repair, being strongly expressed in pyloric metaplasia in Crohn’s disease [22]. Thus, NGAL has properties making it different from calprotectin. Still, its utility for clinical practice in other gut disorders has not been investigated and the mucosal expression of NGAL in CC has not previously been established.

This study aims to comprehensively characterize NGAL/*LCN2* expression in active CC, during response to budesonide treatment and in non-responding patients. We also evaluated its utility as a faecal biomarker of active disease and whether expression of NGAL/*LCN2* may aid in CC histopathological diagnosis.

## Materials and methods

### Clinical material

Patients and healthy volunteers were recruited from Linköping University Hospital, Linköping [25, 26] and St. Olav’s University Hospital, Trondheim. In brief, colonoscopy biopsies were sampled from active CC without treatment (aCC), budesonide-treated CC (9 mg/day, 6–8 weeks) in clinical remission (tCC), and budesonide-refractory CC after discontinuing a prolonged treatment without response (rCC). For some, paired biopsies were taken both before and during response to treatment (aCC-tCC). Healthy controls (HC) had gastrointestinal symptoms and underwent medical assessment concluding with no macroscopic or microscopic disease. Biopsies of active ulcerative colitis (aUC) from our NTNU/St Olav’s University Hospital biobank were used for comparison due to the known increase of NGAL/*LCN2* [23]. The median Mayo Endoscopic Subscore (MES) for UC patients included were 3, range (1–3). All CC and UC biopsies were from the descending colon and control samples from both the hepatic flexure and the descending colon. Biopsies were preserved on 4% formaldehyde or paraformaldehyde for histology, and on Allprotect (Qiagen, Hilden, Germany) (aCC, tCC, rCC and 9 HC) or RNAlater (Sigma-Aldrich, St. Louis, MO) (aUC and 4 HC) for RNA sequencing. Histopathological criteria for CC were thickened subepithelial collagen layer ≥ 10 µm and increased lymphoplasmacytic cell infiltrate in lamina propria. Histopathological criteria for UC were according to current guidelines [27]. Faecal samples were collected prior to endoscopy for some of the same patients and more and stored without additives at − 80 °C. Clinically active disease (aCC) was defined as three or more bowel movements/day or at least one watery bowel movement/day during a 1-week registration period. Clinical remission (tCC) was defined as less than three bowel movements/day and no watery bowel movement during a 1-week registration, both according to Hjortswang criteria [6]. Faecal samples and biopsies were also collected from patients with diarrhoea-predominant IBS, fulfilling ROME III criteria, [28] and with negative colonoscopy and histopathological findings. Most of the patients had samples examined by more than one analysis. Patient and sample details for the different analyses are given in Table 1. All patients gave written informed consent. Ethical approvals were obtained from (Norway) the Central Norway Regional Committee for Medical and Health Research Ethics, 2013/212/REKmidt and (Sweden) Linköping’s Regional Ethical Committee (Dnr. 2015/31-31).

**Table 1:** Patient demographics and number of samples for the RNA sequencing, molecular pathology and faecal analysis

	aCC	tCC	rCC	IBS-D	HC	aUC
^**#**^Subjects, *n*	41	21	14	18	41	17
Gender, *Female* (Male)	34 (7)	17 (4)	13 (1)	15 (3)	25 (16)	10 (7)
Age, *Median* (range)	60 (27–88)	55 (27–86)	56 (25–79)	41 (16–69)	47 (22–71)	30 (17–48)
On 9 mg/kg budesonide	No	Yes	No	–	–	–
Defecation frequency, *median* (range)	7 (3–15)	1 (1–2)	8 (4–16)	^£^–	–	7 (2–7)
^*^RNAseq Samples, *n*	9	9	9	–	13	4
IHC samples, *n*	25	17	14	6	25	12
ISH samples, *n*	22	15	13	–	15	11
Faecal ELISA samples, *n*	28	11	3	14	15	9

*aCC* untreated active collagenous colitis, *tCC* 9 mg/day budesonide-treated collagenous colitis, *rCC* untreated budesonide-refractory collagenous colitis, *IBS-D* diarrhoea dominant functional irritable bowel-syndrome, *HC* healthy control, *IHC*  immunohistochemistry, *ISH* in situ hybridization, *calpro* = calprotectin, *NGAL* neutrophil-gelatinase-associated lipocalin

^#^Within each group of subjects, the majority of samples have 2 or more analyses, except for 45 and 19% of the samples in HC and IBS-D, respectively

^£^All followed the ROME III diagnostic criteria and had diarrhoea as dominating symptom

*All aCC, tCC, rCC and 9 HC samples were stored in Allprotect; aUC and 4 HC samples were stored in RNAlater

### RNA sequencing

Total RNA was extracted from colonic pinch biopsies, processed and analysed as described [25]. In brief, RNA was extracted using RNeasy mini kit (Qiagen) and further quality assessed using Agilent RNA 6000 Pico kit in a 2100 Bioanalyzer (Agilent technologies, CA, USA). Sequencing libraries (SENSE totalRNA with RiboCop rRNA depletion, Lexogene, GmbH, Austria) were single-read sequenced on a NextSeq 500 instrument (Illumina, CA) for 75 cycles to a depth of 25 million base reads pr. sample. Data analysis was done in R v3.5.1, using SARtools v1.6.6 and DESeq2 v1.22.1 packages [29–31]. Test for differential expression was corrected for multiple comparison using Benjamini–Hochberg FDR adjustment, and considered significant if FDR-adjusted *P* value < 0.05. The transcriptome analysis is available at GSE159010 (URL: https://www.ncbi.nlm.nih.gov/geo/query/acc.cgi?acc=GSE159010) [26].

### Histology, immunohistochemistry and in-situ hybridisation

Fixed and paraffin-embedded sections underwent standard pre-treatments for immunohistochemistry (IHC) before incubation with primary antibodies; anti-human NGAL diluted 1:1000 (#44058, Cell Signaling Technology, Inc., Danvers, MA) or anti-human S100A8 (calprotectin) diluted 1:10 000 (MAB4570, R&D Systems, Inc., Bio-Techne, Minneapolis, MN), the secondary antibody EnVision-HRP/DAB + kit (#K5007, Dako Agilent, Santa Clara, CA) and counterstaining with hematoxylin. Non-immunized IgG was used as negative control. All groups including positive control (aUC) were analysed in parallel. In situ hybridization (ISH) was performed using the RNAscope 2.5 HD Reagent Kit (Brown) (#322300), a human *LCN2* probe (#559441, Advanced Cell Diagnostics, Inc. (ACD), Bio-Techne) and counterstaining with hematoxylin. Slides were digitalized using NanoZoomer S360 (Hamamatsu, Hamamatsu City, Japan) Images were captured using the export function in NDP.view2 software (U12388-01, Hamamatsu) or by Nikon E400 microscope, DS-Fi1 camera and NIS-Elements BR imaging software (Nikon Corporation, Tokyo, Japan). Further processing was done using Photoshop (Adobe Photoshop CC, 20.0.6 Release) and Fiji [32]. For a semi-quantitative assessment of the tissue stains, variants of common single or composite ordinal scores were used [33] and based on whole-slide images of all the tissue in each biopsy. Scoring of the epithelial expression was for IHC (NGAL) done by multiplying maximum epithelial staining intensity (0 = no, 1 = weak, 2 = moderate, 3 = strong) with staining distribution (1 = focal, 2 = widespread), while for ISH (*LCN2* mRNA), the ACD scoring system (0 = 0 dots/cell, 1 = 1–3 dots/cell, 2 = 4–9 dots/cell and none/very few dot clusters, 3 = 10–15 dots/cell and/or > 10% dots in cluster, 4 =  > 15 dots/cell and/or > 10% dots in cluster) [34] was used, multiplied with staining distribution (1 = focal, 2 = widespread). Mucosal proportion of calprotectin-expressing cells was subjectively estimated as 1 (< 2%), 2 (2–19%), 3 (20–39%) or 4 (> 40%). Samples were evaluated blinded for group by two independent observers (IB, AKS). Routine H&E sections were examined for histopathological features relevant to CC (chronic inflammation (lymphocyte and plasma cell infiltration in lamina propria), subepithelial collagen band, surface epithelium flattening and detachment, intraepithelial lymphocytes (IEL) in surface or crypt epithelium, eosinophils, architecture, Paneth cells, neutrophils in lamina propria and crypts and erosion), and graded as No = 1 (no histological abnormalities), Mild = 2 (subtle histological changes) or Yes = 3 (pronounced histological changes); and correlated to the epithelial NGAL IHC scores in HC and CC.

### NGAL and calprotectin analyses in faeces

Faecal samples were blinded to clinical status and analysed for NGAL and calprotectin by enzyme-linked immunosorbent assay (ELISA). For NGAL, stool samples were diluted 1:5 (w/v), centrifuged and diluted to 1:500 and 1:1000 (1:6000 for aUC) (DY1757, R&D systems). Four samples (1 tCC, 1 IBS-D, 2 HC) were below range and set to half the detection limit (0.1 mg/kg). Calprotectin was analysed from the same faecal samples, diluted 1:50 (w/v) in Calpro Easy Extract by Calpro AS (Lysaker, Norway). Forty (5 aCC, 8 tCC, 15HC, 12 IBS-D) samples were below range and set to half the detection limit (12.5 mg/kg).

### Statistics

Statistical analyses (other than sequencing data) were performed with GraphPad Prism 8 (GraphPad software, San Diego, CA). All datasets were analysed with the nonparametric Kruskal–Wallis test and Dunn’s multiple comparisons post-test, Wilcoxon matched-pairs signed rank test for paired data or Spearman correlation coefficient. *P* value < 0.05 was considered significant and confidence interval (CI) was 95%. Sensitivity and specificity of the faecal ELISAs were calculated as receiver operating characteristic (ROC) curves and area under the curves (AUC).

## Results

### Expression of *LCN2* mRNA is increased in colonic mucosa of aCC

**Fig. 1:**
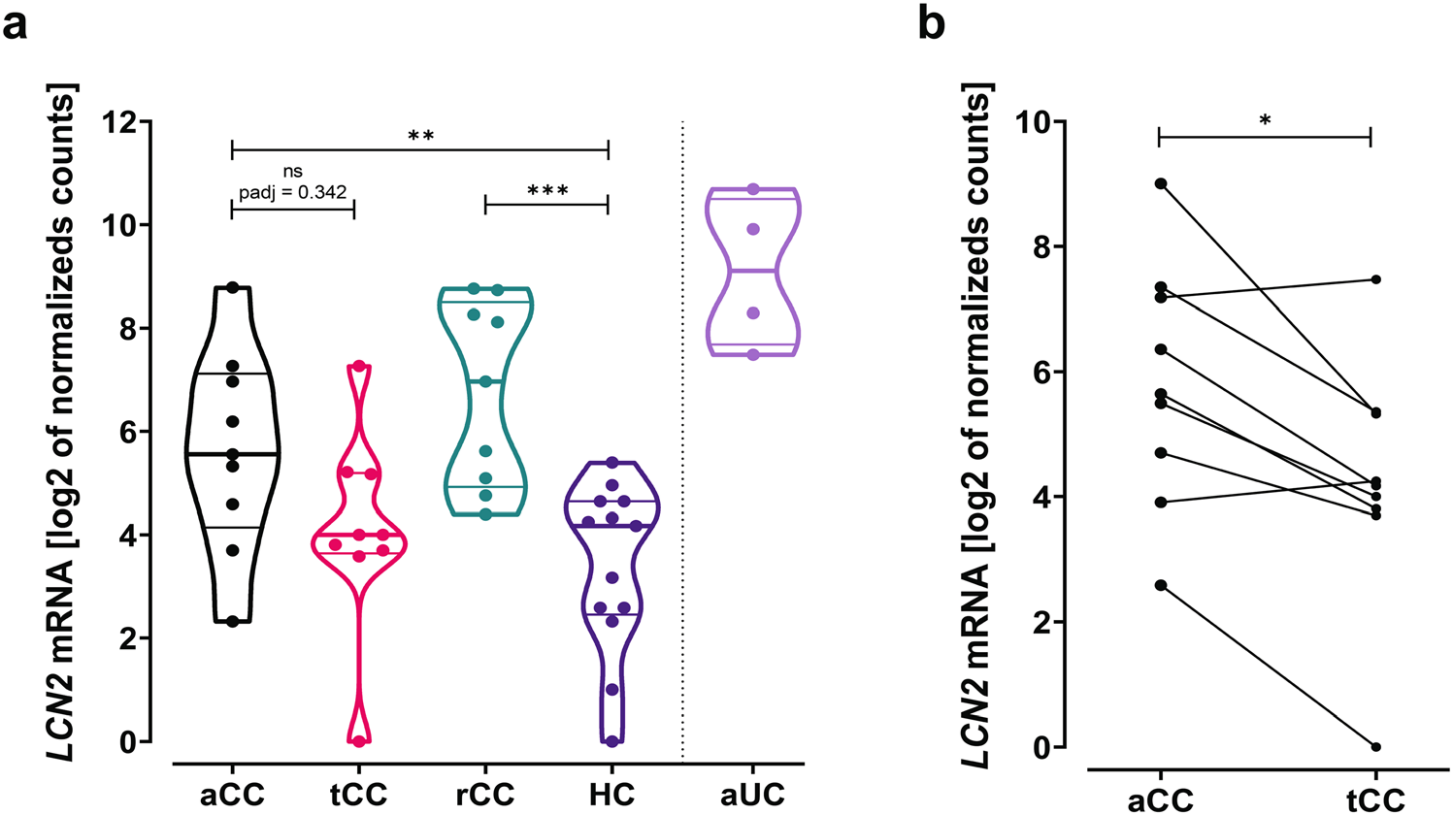
RNA sequencing of *LCN2* mRNA in colonic mucosa of collagenous colitis. **a** *LCN2* mRNA expression in pinch biopsies from colonic mucosa of active collagenous colitis (aCC), budesonide-treated collagenous colitis with clinical remission (tCC), budesonide-refractory collagenous colitis (rCC) and healthy controls (HC). Active ulcerative colitis (aUC) was included for comparison (separated by dotted line). The y-axis shows *LCN2* mRNA expression as log2 of normalized counts and the violin plots visualize distribution frequency in addition to individual values (dots), median (thick line), upper and lower quartiles (thin lines). **b** Paired analysis of *LCN2* mRNA expression in colonic mucosa of individual patients with active collagenous colitis before (aCC) and during budesonide treatment with clinical remission (tCC). The y-axis shows *LCN2* mRNA expression as log 2 of normalized counts in paired biopsies from nine individual patients. P adj = adjusted P value, ns = non-significant, *P < 0.05, ***P < 0.001, ****P < 0.0001 Analysed as described in “Materials and methods”

RNA sequencing analysis of *LCN2* was drawn from an already available dataset providing a full transcriptomic profiling of colonic mucosa from patients with CC (available at GSE159010) [25, 26]. The results showed a variable but upregulated *LCN2* in patients with aCC compared to HC (fold change 6.47, log2 fold change 2.69, *P* = 0.005) (Fig. 1a). Patients in clinical remission during treatment (tCC) displayed *LCN2* expression similar to controls. Paired analyses of aCC and tCC showed that budesonide downregulated the *LCN2* expression (fold change 0.35, log2 fold change − 1.54, *P* = 0.042) (Fig. 1b). Budesonide-refractory patients (rCC) had increased *LCN2* expression compared to controls (fold change 11.94, log2 fold change 3.58, *P* < 0.0001), comparable to aCC. The aUC samples included as positive control confirmed a pronounced increase in *LCN2* expression [23] (Fig. 1a).

### Increased expression of NGAL protein and *LCN2 *mRNA in aCC is localized to the epithelium

**Fig. 2:**
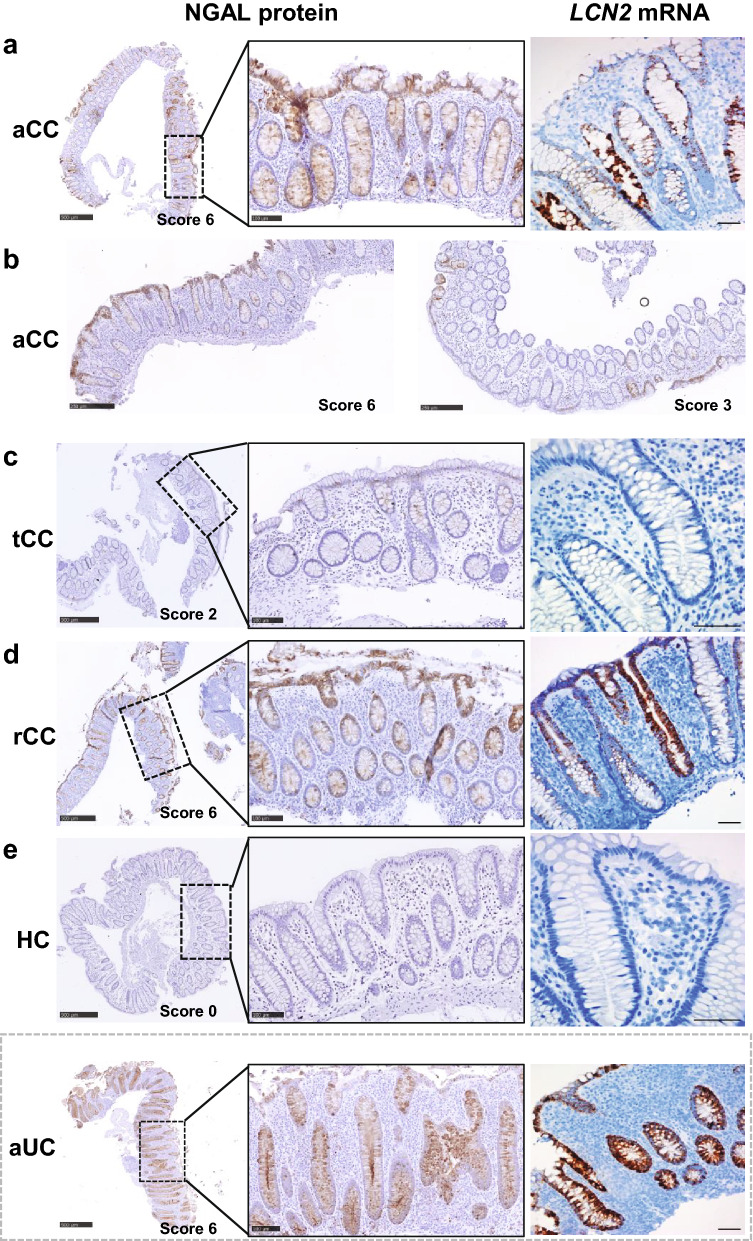
Representative images of IHC and ISH showing expression of NGAL protein and *LCN2* mRNA in colonic epithelium in collagenous colitis**. a** Overview of NGAL protein expression (left panel), with higher magnification of epithelial staining throughout the crypts (middle panel), and *LCN2* mRNA expression (right panel) in colonic mucosa of patients with active collagenous colitis (aCC). **b** Overview of NGAL expression in aCC mostly in the surface epithelium (left panel) or with a patchy appearance (right panel) **c**–**e** Overview of NGAL protein expression (left panel), with higher magnification as indicated (middle panel), and *LCN2* mRNA expression (right panels) in colonic mucosa of patients with budesonide-treated collagenous colitis in clinical remission (tCC), **d** budesonide-refractory collagenous colitis (rCC) and **e** healthy controls (HC). Active ulcerative colitis (aUC) was included for comparison (separated by dotted grey frame). The IHC NGAL scores are given for each image. Scale bars 500 µm (left panel), 100 µm (middle panel), 50 µm (right panel)

ISH for *LCN2* and IHC for NGAL confirmed increased mucosal expression in aCC for mRNA and protein and identified epithelial cells as the main source (Fig. 2a, b). During budesonide-induced remission (tCC), the expression was markedly reduced and close to the level in HC (Fig. 2c, e), while the mucosa in budesonide-refractory disease (rCC) displayed an expression similar to aCC (Fig. 2d). In contrast, the epithelial expression of *LCN2*/NGAL was almost absent in HC (Fig. 2e), and strongly increased throughout the colonic crypts in aUC as previously reported [16, 23]. Although some of the aCC and rCC samples also showed epithelial *LCN2*/NGAL expression throughout the crypts (Fig. 2a, d), there were generally more samples with predominant expression in the surface epithelium (Fig. 2b, left panel) and with a patchy appearance (Fig. 2b, right panel) compared to aUC.

**Fig. 3:**
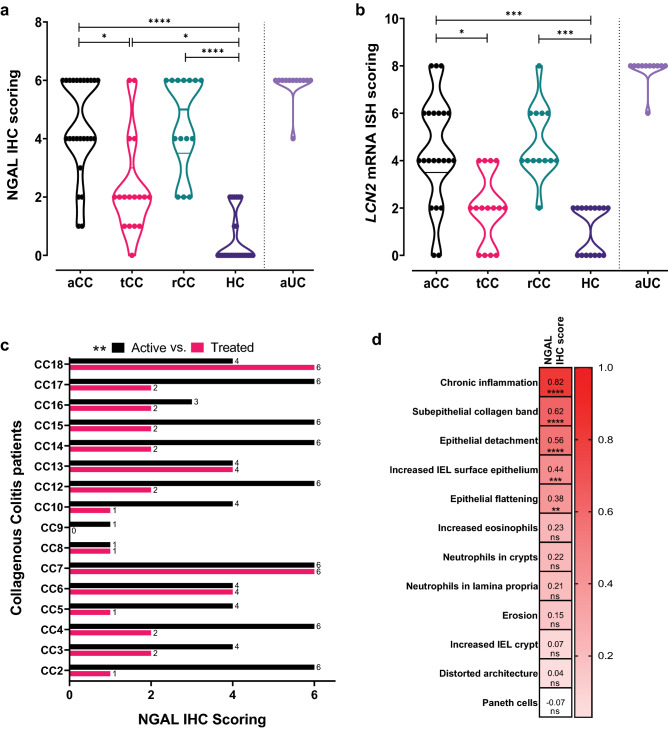
Semi-quantitative scoring of the colonic epithelial expression levels of NGAL protein and *LCN2* mRNA analysed by IHC and ISH staining and correlation to relevant histopathological features. **a**, **b** Total score was achieved by multiplying maximum epithelial staining intensities with staining distribution for (**a**) NGAL IHC and (**b**) *LCN2* mRNA ISH in colonic mucosa of patients with active collagenous colitis (aCC), budesonide-treated collagenous colitis with clinical remission (tCC), budesonide-refractory collagenous colitis (rCC) and healthy controls (HC). Active ulcerative colitis (aUC) was included for comparison (separated by dotted line). The violin plots visualize distribution frequency in addition to individual values (dots), median (thick line), upper and lower quartiles (thin lines). *P < 0.05, ***P < 0.001, ****P < 0.0001 analysed with Kruskal–Wallis with Dunn’s multiple comparisons test. **c** Paired analysis of the NGAL IHC epithelial scores in individual patients (CC2–CC18) with active collagenous colitis before (aCC) and during budesonide treatment with clinical remission (tCC). **P < 0.01 analysed with Wilcoxon test. **d** Heatmap showing correlation of the epithelial NGAL IHC scores in the CC groups, HC and IBS-D to different histopathological features relevant to the diagnosis of CC. Spearman correlation coefficient r and *P < 0.05, **P < 0.01, ***P < 0.001, ****P < 0.0001 or *ns* non-significant are given for all the different features

A semi-quantitative scoring was used to obtain a more objective assessment of the *LCN2* and NGAL staining (Fig. 2). Although there was noticeable variability within the CC groups (more than in HC and aUC) (Fig. 3a, b), the scores were higher in aCC for both *LCN2* and NGAL compared to HC (*P* = 0.0003 and *P* < 0.0001, respectively) and tCC (*P* = 0.019 and *P* = 0.029, respectively), and in rCC compared to HC (*P* = 0.0004 and *P* < 0.0001, respectively). The *LCN2* scores in tCC were similar to HC (Fig. 3b), but the reduced NGAL scores in tCC did not reach HC level (*P* = 0.036) (Fig. 3a). Paired analyses of aCC and tCC confirmed a decrease in NGAL scores from active disease to remission for 14 out of 18 patients (*P* = 0.0024) (Fig. 3c).

Due to these novel findings, we performed a preliminary study with the other MC subtypes. Both NGAL IHC and *LCN2* ISH on colonic mucosal samples from patient with active lymphocytic colitis (aLC) (*n* = 3) and incomplete MC (MCi) (*n* = 3) showed high epithelial expression similar to aCC (See Supplementary Fig. 1 showing images and patient characteristics).

### NGAL/*LCN2* levels correlate with histopathological changes and stool frequency in CC

To further elucidate histopathological and clinical significance of the NGAL scores, we comprehensively characterized the H&E staining histology and recorded stool frequency. In aCC and rCC typical changes were seen, like subepithelial collagen deposition, epithelial flattening and detachment, and chronic inflammation. Despite clinical remission in tCC, the histology still resembled the samples from patients with active disease (Supplementary Table 1 showing features in aCC, tCC and rCC). Interestingly, the NGAL scores in all patient groups were generally higher the more histopathological features present (Supplementary Table 2 showing NGAL IHC scores for all groups correlated to histology). In the HC and CC groups, epithelial NGAL expression most strongly correlated to the degree of chronic inflammation (*r* = 0.817, 95% CI 0.7097–0.8870, *P* < 0.0001) showing higher IHC scores the higher degree of chronic inflammation (Supplementary Table 2). Also subepithelial collagen deposition (*r* = 0.619, CI 0.4325–0.7548, *P* < 0.0001), epithelial detachment (*r* = 0.556, CI 0.3513–0.7104, *P* < 0.0001), number of surface IELs (*r* = 0.436, CI 0.1992–0.6242, *P* < 0.001) and epithelial flattening (*r* = 0.382, CI 0.1360–0.5829, *P* < 0.01) correlated with higher NGAL IHC scores (Fig. 3d, Supplementary Table 2). As expected, NGAL IHC and histological evaluation of IBS-D patients (*n* = 6) showed results similar to HC (Supplementary Table 2 showing NGAL IHC scores and histology, and Supplementary Fig. 2 showing IHC images).

Furthermore, the principal clinical metric for CC disease activity, stool frequency, correlated with both NGAL (*r* = 0.4232, CI 0.1700–0.6239, *P* = 0.0013) and *LCN2* scores (*r* = 0.3646, CI 0.0876–0.5892, *P* = 0.0092).

### Different cellular localization of calprotectin and NGAL in active CC

**Fig. 4:**
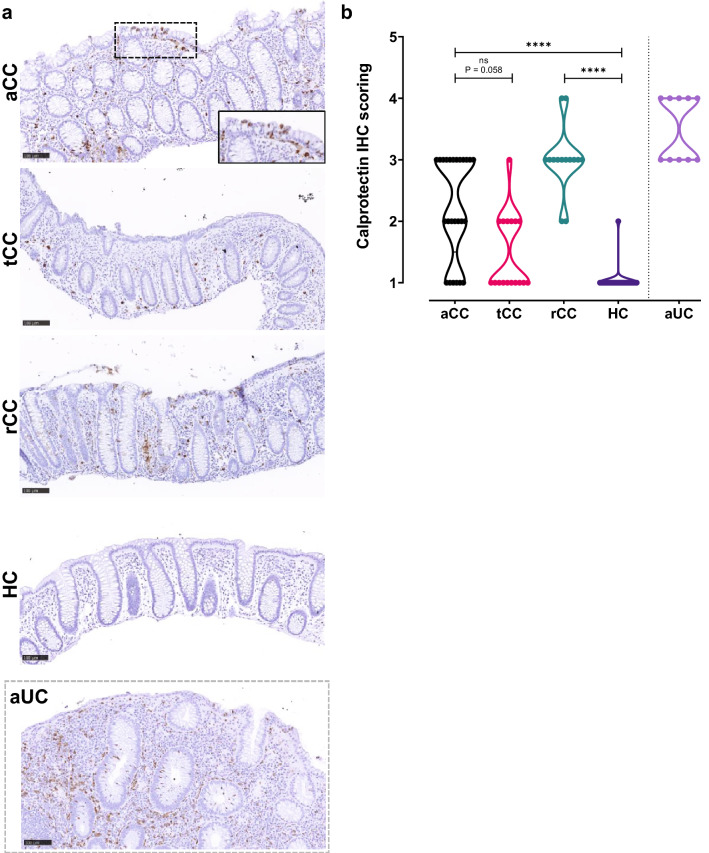
Calprotectin in colonic mucosa of collagenous colitis. **a** Representative images of IHC showing calprotectin protein expression in the colonic mucosa of patients with active collagenous colitis (aCC), budesonide-treated collagenous colitis with clinical remission (tCC), budesonide-refractory collagenous colitis (rCC) and healthy controls (HC). Higher magnification image of the surface epithelium in inset as indicated. Active ulcerative colitis (aUC) was included for comparison (separated by dotted grey frame). Scale bars 100 µm. **b** Semi-quantitative scoring of calprotectin-expressing cells in the colonic mucosa of patients in (**a**). The violin plot visualizes distribution frequency in addition to individual values (dots), median (thick line), upper and lower quartiles (thin lines). Active ulcerative colitis (aUC) was included for comparison (separated by dotted line). *ns* non-significant, ****P < 0.0001 analysed with Kruskal–Wallis with Dunn’s multiple comparisons test

The histological pattern of calprotectin in mucosa of CC has not been described previously, thus we compared this established biomarker for IBD with NGAL/*LCN2*. Staining identified more calprotectin-expressing immune cells in the lamina propria of aCC and rCC compared to HC (Fig. 4), in some cases these cells were also seen infiltrating between the epithelial cells. In contrast to NGAL, calprotectin was not expressed in the colonic epithelial cells (Fig. 4a, inset). Correspondingly, also a slight increase in NGAL-expressing immune cells was found in the lamina propria of aCC and rCC (Fig. 3). Since the CC samples showed almost no infiltrating neutrophils (See Supplementary Tables), these immune cells probably represent myeloid cells expressing minor amounts of NGAL and/or calprotectin [21, 35]. In contrast, aUC had massive infiltration of acute inflammatory neutrophils expressing both NGAL and calprotectin. The semi-quantitative scoring confirmed higher proportion of calprotectin-expressing cells in aCC (*P* < 0.0001) and rCC (*P* < 0.0001) compared to HC, although not as high as in aUC, with a trend towards reduction during budesonide treatment (tCC) (*P* = 0.058) (Fig. 4b).

### Faecal NGAL and calprotectin levels are increased in active CC

**Fig. 5:**
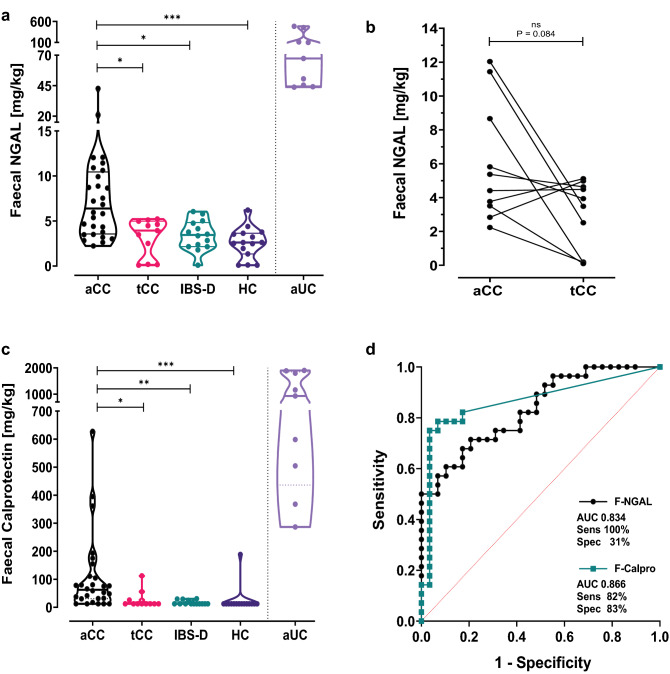
Faecal NGAL and calprotectin in collagenous colitis. **a** NGAL in faeces measured by ELISA in samples from patients with active collagenous colitis (aCC), budesonide-treated collagenous colitis with clinical remission (tCC), diarrhoea dominant functional irritable bowel-syndrome (IBS-D) and healthy controls (HC). The y-axis shows the concentration of NGAL in mg per kg faeces. *P < 0.05, ***P < 0.001 analysed with Kruskal–Wallis with Dunn’s multiple comparisons test. **b** Paired analysis of the faecal NGAL concentrations in individual patients with active collagenous colitis before (aCC) and during budesonide treatment with clinical remission (tCC). The y-axis shows the concentration of NGAL in mg per kg faeces in paired samples from 10 individual patients. *ns* non-significant analysed with Wilcoxon test. **c** Calprotectin in faeces measured by ELISA in samples from patients in (**a**). The y-axis shows the concentration of calprotectin in mg per kg faeces. The violin plots visualize distribution frequency in addition to individual values (dots), median (thick line), upper and lower quartiles (thin lines). Active ulcerative colitis (aUC) was included for comparison (separated by dotted line). *P < 0.05, **P < 0.01, ***P < 0.001 analysed with Kruskal–Wallis with Dunn’s multiple comparisons test. **d** Receiver operating characteristic (ROC) curves of ELISA faecal NGAL (F-NGAL) (black line) and calprotectin (F-calpro) (green line) when comparing aCC against HC and IBS-D. Area under the curve (AUC), and maximal sensitivity (sens) and specificity (spec) when using cut-off values 2.2 mg/kg for F-NGAL and 14.85 mg/kg for F-calpro are given. The red dotted line shows line of identity

To further validate NGAL/*LCN2* as a non-invasive biomarker for CC in clinical practice, we analysed faecal NGAL (F-NGAL) and compared to faecal calprotectin (F-calpro). We found that the increased mucosal expression of NGAL was clearly reflected in stool. F-NGAL (mg/kg) was elevated in aCC compared to HC (*P* = 0.0001) and to IBS-D (*P* = 0.011) (Fig. 5a), while F-NGAL concentrations in HC and IBS-D were similar and as shown by us previously [16]. As in the other analyses, there was a striking variability between patients with aCC, with some having clearly elevated F-NGAL concentrations compared to HC and IBS-D while others had overlapping values. On a group level, F-NGAL was reduced during budesonide treatment (tCC) compared to aCC (*p* = 0.037) (Fig. 5a), while paired analysis of aCC and tCC showed a clear trend towards reduction (*P* = 0.084) (Fig. 5b). Faecal samples were available from only 3 rCC patients and the F-NGAL concentrations varied extensively (data not shown). Thus, further investigation of this group is required. Interestingly, as for the NGAL/*LCN2* scores, F-NGAL concentrations in CC patients correlated with stool frequency (*r* = 0.4466, CI 0.1431–0.6733, *P* = 0.0044). ROC curve analyses to evaluate how the F-NGAL test performed in separating patients in need of a diagnostic colonoscopy (aCC) from those who do not (HC and IBS-D) showed an AUC of 0.834 (CI 0.732–0.936). Using a cut-off value of 2.2 mg/kg, the F-NGAL test performed with 100% sensitivity detecting all aCC patients, but with concurrent specificity of 31% (Fig. 5d).

The F-calpro values (median 62.4, range 12.5–627.7) in aCC patients were mainly below 100 mg/kg, but significantly higher than HC (*p* = 0.0003), IBS-D (*p* = 0.0028) and tCC (*p* = 0.019). Still, F-calpro values in aCC were much lower than in aUC (Fig. 5c). ROC curve analyses of F-calpro performance in separating those with aCC from HC and IBS-D showed an AUC of 0.866 (CI 0.762–0.970). Maximal test sensitivity of 82% with a specificity of 83% was reached using cut-off 14.85 mg/kg. Increasing the cut-off value to a more clinically relevant number of 50.4 mg/kg, reduced sensitivity to 57% and maintained a specificity of 97% (Fig. 5d).

## Discussion

Mucosal overexpression of NGAL/*LCN2* in inflamed colonic epithelium of active CC, which is also reflected in elevated levels of NGAL in feces, are novel findings that can be useful in clinical practice. Chronic diarrhoea is frequent, has many causes and can be challenging to diagnose. Our results pinpoint F-NGAL as a potential biomarker selecting patients suffering from chronic diarrhoea for colonoscopy to ensure diagnosis of active CC. In contrast to classic IBD, the endoscopic findings in CC are subtle and diagnosis depends on a high level of suspicion and multiple biopsies. Formal criteria for routine histopathological diagnosis of CC exist [4], but clinical decision can be challenging, especially with diffuse clinical information, poorly oriented biopsies or mild changes. The incomplete MC form is even more difficult, and our preliminary results suggest overexpression of NGAL/*LCN2* also in the other MC subtypes. Thus, IHC and ISH for NGAL/*LCN2* in a mucosa which appears normal on routine histology, may provide pathologists with an additional diagnostic tool.

Our results indicate activation of the epithelium and align well with previous observations in classic IBD [16, 36], which could share disease mechanisms with CC involving the epithelial cells. In aCC we found NGAL often expressed mainly in the luminal epithelium *versus* aUC where NGAL is found throughout the crypts. aUC is characterized by neutrophil infiltration. Neutrophils release proinflammatory cytokines that are shown to induce NGAL in vitro colonic epithelial cells and may explain the expression of NGAL throughout the crypts. Since neutrophils inconsequently are found in aCC, the NGAL expression in luminal epithelium may result from more direct induction. For example through luminal derived ligands of Toll-like receptors TLR3 and TLR5 in the epithelium, which are innate immune sensors known to induce NGAL in colonic epithelial cells 23, 37, 38. Barrier dysfunction and increased luminal influx has been described in active MC [39] and previous observation has connected the increased number of IELs to an unknown luminal agent central in the development of CC [40, 41]. In addition, NGAL’s bacteriostatic activity [20] could contribute to dysbiosis in CC [42]. Since intestinal epithelium is an important source of cytokines and contributes to bacteria-mediated immunomodulation in classic IBD [43], a similar role for the epithelium in CC is possible. On the other side, the overt destructive inflammation in aUC causes a more intense regeneration and proliferation of epithelial cells than in the non-destructive colitis features observed in aCC. Indeed, NGAL is also involved in proliferation and regeneration of intestinal epithelium [22]. Thus, this might represent an alternative explanation of NGAL expression in crypts (aUC) compared to the surface epithelium (aCC). In addition, NGAL expression is observed in several other inflammatory bowel conditions like infectious gastroenteritis [16, 44]. This warrants the question whether NGAL expression could be a potential marker for early epithelial activation and inflammation development. Altogether, exactly how NGAL relates to the inflammatory activity of CC remains unclear and would need further mechanistic studies.

Epithelial NGAL/*LCN2* expression was normalized during budesonide treatment leading to cessation of diarrhoea, and NGAL/*LCN2* scores and F-NGAL levels correlated with stool frequency. Contrary, even if typical histological features of CC, like abnormal collagen band, epithelial injury and increased surface IEL, also correlated to the NGAL score these were still evident in the biopsies taken during treatment-induced remission. The trait most strongly correlating to the amount of epithelial NGAL was degree of chronic inflammation in lamina propria, more than the thickened collagen band required for CC diagnosis. Thus, NGAL/*LCN2* expression seems to reflect clinical symptoms and remission, while the histopathological changes do not [15]. Interestingly, lamina propria inflammation is an unspecific feature of many conditions and frequent in biopsies prior (within 1 year) to appearance of the histological hallmarks and diagnosis of MC in patients with chronic watery diarrhoea [45]. Increased NGAL/*LCN2* expression appears to reflect activation of the epithelium, which could be a primary event in CC pathophysiology leading to diarrhoea, and is therefore a potential biomarker for disease activity in CC. Currently, there are no established metrics to measure disease activity besides the Hjortwang criteria for disease remission [6]. Refractory CC or CC relapse is frequent, and we foresee a discussion of treatment target in CC that parallels the still ongoing discussion in IBD. The previous treatment target of clinical remission in IBD is now challenged by an endoscopic or even histopathological definition of remission, which also reflect a reduced risk of relapse. Since the histopathological findings of aCC do not dissolve during clinical remission, NGAL may be a better marker for an activated colonic epithelium and hence for ongoing mucosal inflammation. We thus propose that IHC for NGAL in colonic biopsies may improve decision making in cases of chronic diarrhoea and treatment refractory CC.

F-NGAL in active IBD originates from both constitutive expression in invading neutrophils and strong induction in intestinal epithelial cells [23], while F-NGAL in active CC mostly reflects colonic epithelial upregulation. Thus, the NGAL protein exhibits features that make it particularly useful to assess disease activity in CC and superior to F-calpro, which is a good biomarker for active classic IBD but depends on a neutrophil infiltration not seen in MC [18, 19, 46]. The main infiltrating immune cells in CC are lymphocytes, plasma cells, and other types of the myeloid lineage where NGAL expression is broader than calprotectin [21], giving F-NGAL a further advantage as a biomarker for MC compared to calprotectin.

Here, we do find significantly increased F-NGAL in aCC compared to HC and IBS-D and the levels decreased upon successful budesonide treatment (tCC), correlating to stool frequency. Notably, the broad distribution in aCC and the partial overlap with HC and IBS-D suggest disease subgroups, with possible therapeutic implications and/or significant pathobiological diversity within this diagnosis. Since the optimal clinical cut-off value for F-NGAL in CC is still largely unexplored, a larger sample size is needed to conclude and to set a proper value for CC. More thorough and systematic clinical characterisation and evaluation of biopsies is needed for subgroup comparisons. Thus, ROC curve analyses might be preliminary, but a relatively good AUC and 100% sensitivity show that the F-NGAL test is able to detect all aCC patients that are in need of a colonoscopy to confirm their diagnosis and get correct treatment. The lower specificity might lead to unnecessary colonoscopies, but in daily clinical practice, the most important aspect could be to detect those not in risk of having aCC and not in need of endoscopy.

We largely reproduce previous findings that F-calpro is increased in aCC but with considerable overlap with tCC, HC and IBS-D [4, 18, 19, 46]. Many of the F-calpro values were in the 50–100 mg/kg borderline range for inflammation using the Calpro ELISA kit, and below what is commonly considered clinically important. Although the correct cut-off for F-calpro in CC is also still unknown and probably lower than for classic IBD, the test sensitivity did not surpass 83% using a very low cut-off value. Increasing cut-off to 50 mg/kg decreased sensitivity further to 57%. Thus, our results support the clinical consensus that many MC patients remain undetected when using F-calpro, while F-NGAL so far seems superior for detecting aCC patients.

As an explorative study, our sample size is relatively small but enough to highlight the relevance of NGAL and its use in clinical practice as a valuable biomarker for CC. Another possible limitation of the study is a higher mean age in the aCC group compared to HC, opening for a relationship between NGAL and age. However, in sub-analyses, we do not find any correlation between NGAL levels and age within any groups (data not shown). Due to challenging rCC diagnose and sampling, we only had 3 faecal samples available. However, in rCC biopsies we show increased expression of *LCN2* mRNA and strong NGAL staining comparable to aCC, which support NGAL as a biomarker for rCC as well. On this background we expect F-NGAL to be increased in rCC patients, but this remains to be proven.

To conclude, we identify increased epithelial NGAL as a putative tool in the histopathological diagnosis of CC, and as the hitherto most promising faecal biomarker for this disease. Although more work is needed to assess its utility, our observations identify NGAL as a novel element in CC pathobiology, a factor which should be further explored to improve our understanding of this poorly characterized disease.

## Supplementary Information

Below is the link to the electronic supplementary material.

Supplementary file1 (PDF 8196 KB)

Supplementary file2 (PDF 7070 KB)

Supplementary file3 (XLSX 24 KB)

## Abbreviations

aCC: Active collagenous colitis
aUC: Active ulcerative colitis
CC: Collagenous colitis
CD: Crohn's disease
ELISA: Enzyme-linked immunosorbent assay
F-calpro: Faecal calprotectin
F-NGAL: Faecal neutrophil gelatinase-associated lipocalin
FFPE: Formalin-fixed, paraffin-embedded
HC: Healthy control
IBD: Inflammatory bowel disease
IBS-D: Diarrhoea dominant functional irritable bowel-syndrome
IEL: Intraepithelial lymphocytes
IHC: Immunohistochemistry
ISH: In situ hybridization
LC: Lymphocytic colitis
Lcn2: Lipocalin 2
MC: Microscopic colitis
NGAL: Neutrophil gelatinase-associated lipocalin
rCC: Refractory collagenous colitis
RNAseq: Whole transcriptome shotgun sequencing
tCC: Treated collagenous colitis
TLRs: Toll-like receptors
